# Simvastatin induces pyroptosis via ROS/caspase-1/GSDMD pathway in colon cancer

**DOI:** 10.1186/s12964-023-01359-y

**Published:** 2023-11-16

**Authors:** Wei Xie, Mingjing Peng, Ying Liu, Bocheng Zhang, Liang Yi, Ying Long

**Affiliations:** 1 Translational medicine centre, Hunan Cancer Hospital and the Affiliated Cancer Hospital of Xiangya School of Medicine, Central South University, Changsha, 410013 Hunan P. R. China; 2 Department of Hepatobiliary Surgery, Hunan Cancer Hospital and the Affiliated Cancer Hospital of Xiangya School of Medicine, Central South University, Changsha, 410013 Hunan P. R. China; 3 Central laboratory, Hunan Cancer Hospital and the Affiliated Cancer Hospital of Xiangya School of Medicine, Central South University, Changsha, Hunan 410013 P. R. China; 4 Hunan Provincial Clinical Research Centre for Oncoplastic Surgery, Hunan Cancer hospital and the Affiliated Cancer Hospital of Xiangya School of Medicine, Changsha, Hunan 410013 P. R. China

**Keywords:** Simvastatin, Caspase-1, Pyroptosis, ROS, Colon cancer

## Abstract

**Background:**

The outcome of patients with colon cancer is still unsatisfied nowadays. Simvastatin is a type of statins with anti-cancer activity, but its effect on colon cancer cells remains unclear. The present study is intended to determine the underlying mechanism of simvastatin in treatment of colon cancer.

**Methods:**

The viability and pyroptosis rate of cells treated and untreated with simvastatin were analysed by CCK-8 and flow cytometry assays, respectively. We used DCFH-DA and flow cytometry to detect reactive oxygen species (ROS) production. Levels of pyroptosis markers were detected by western blotting analysis or immunofluorescence staining. Besides, the anticancer properties of simvastatin on colon cancer were further demonstrated using a cell line based xenograft tumor model.

**Results:**

Simvastatin treatment in HCT116 and SW620 induced pyroptosis and suppressed cell proliferation, with changes in the expression level of NLPR3, ASC, cleaved-caspase-1, mature IL-1β, IL-18 and GSDMD-N. Moreover, inhibition of caspase-1 and ROS attenuated the effects of simvastatin on cancer cell viability. In addition, it was identified that simvastatin has an anti-tumor effect by down-regulating ROS production and inducing downstream caspase-1 dependent pyroptosis in the subcutaneous transplantation tumors of HCT116 cells in BALB/c nude mice.

**Conclusions:**

Our in vitro and in vivo results indicated that simvastatin induced pyroptosis through ROS/caspase-1/GSDMD pathway, thereby serving as a potential agent for colon cancer treatment.

Video Abstract

**Supplementary Information:**

The online version contains supplementary material available at 10.1186/s12964-023-01359-y.

## Introduction

Colon cancer is the fourth leading cause of cancer-related death worldwidely [1]. The incidence of colon cancer has increased over past decades. Despite significant progress in surgery, chemotherapy, radiotherapy, targeted therapy and immunotherapy, many colon cancer patients ultimately face recurrence, metastasis and death. Therefore, it is of great significance for developing novel treatment strategies and drugs to improve the prognosis of colon cancer patients.

Simvastatin was mainly used in the treatment of hyperlipidemia in the past, but recently it has been rediscovered for exhibiting anticancer activities [2]. Increasing evidence indicated that simvastatin affected the proliferation, survival, migration, invasion, chemosensitivity and radiosensitivity of cancer cells [3–7]. In particular, in vitro studies it was highlighted that simvastatin suppressed proliferation and angiogenesis in colorectal cancer [8, 9]. However, the effect and underlying mechanism of simvastatin on colon cancer cells remain to be well elucidated.

Tumor cells with uncontrolled proliferation provide them with growth advantages, and these cells are always characterized by derangements in cell cycle or/and cell death regulation [10–13]. As a type of programmed cell death (PCD), pyroptosis is characterized by the morphology of inflammatory cell death. Increasing studies indicated that pyroptosis played important roles in cancer development and treatment [14–16]. Previous reports had demonstrated that simvastatin suppressed tumor growth through regulating pyroptosis in non-small cell lung cancer and glioblastoma [17, 18]. Additionally, Liu and his colleagues found that expression of pyroptosis-related lncRNAs was associated with prognosis of colon cancer patients [19]. However, the relationship between simvastatin and pyroptosis in colon cancer remains unclear.

In the present study, we attempted to examine the effect of simvastatin on colon cancer and to discover its underlying mechanism. Our results showed that simvastatin inhibited the viability of colon cancer cells by inducing pyroptosis, which might shed new light on the anti-tumor mechanism of this drug.

## Materials and methods

### Reagents and antibodies

CCK-8 kit (C0041) and DCFH-DA staining solution (S0033S) were purchased from Beyotime Biotechnology (Shanghai, China). For western blot assays, PVDF membranes was purchased from Millipore (Billerica, USA), and enhanced chemiluminescence (ECL) kit was obtained from Pierce (Rockford, USA). Fetal bovine serum (FBS) for cell culture was obtained from Gibco (Gaithersburg, USA). Simvastatin (S831014) was purchased from Macklin (Shanghai, China). VX-765 (S2228) and N-acetyl-l-cysteine (NAC, S1623) were obtained from Selleck (Shanghai, China). Primary antibodies against Caspase-1 (22,915), NLRP3 (19,771), IL-18 (10,663), and β-actin (66,009) were purchased from ProteinTech Co. Ltd (Illinois, USA). Primary antibodies against IL-1β (ab254360) and GSDMD (ab209845) were obtained from Abcam Inc. (Cambridge, UK), and primary antibodie against ASC (bs-6741R) was obtained from Beijing Biosynthesis Biotechnology Co., Ltd. (Beijing, China). The HRP-conjugated secondary antibodies (SA00001-1 and SA00001-2) and Alexa Fluor® 488-conjugated secondary antibodies (srbAF488-1) were purchased from ProteinTech Co. Ltd (Illinois, USA). For flow cytometry, active caspase-1 staining kit (ab219935) was purchased from Abcam Inc. (Cambridge, UK).

### Cell lines

HCT116 cell line was a gift from Dr. Longzheng Xia, and SW620 cell line were originally from ATCC and maintained in our lab. HCT116 and SW620 cells were routinely cultured in DMEM, L15, respectively, supplemented with 10% FBS, penicillin (100 U/mL), and streptomycin (100 µg/mL), and incubated at 37 °C. The human colon epithelial cell line, NCM460 (INCELL Corporation, LLC), was a gift from Professor Tianhui Hu of Xiamen University, and cultured as described in the manual? in our lab. Cells were treated with different combination of simvastatin, VX-765 and NAC before the assays start in the further research.

### Cell counting kit-8 (CCK-8) assay

Each group of cells were seeded into 96-well plates at 2 × 10^3^ cells/well, and incubated with a concentration gradient of simvastatin for 24 h. The CCK-8 reagent was pipetted into the well after 0-, 24- and 48-hour of culturing, and the optical density at 450 nm was examined using a microplate reader after a 2-hour incubation.

### Flow cytometry

Pyroptosis of treated and untreated cells was measured by flow cytometry using propidium iodide (PI) and caspase-1 staining. Briefly, each group of cells were harvested by centrifugation at 1000 rpm for 5 min after washing with PBS twice. Then, cells were incubated with caspase-1 antibody and PI for 1 h at room temperature in the dark. The cells were analyzed using a flow cytometry, and the experiment was repeated at least three times.

### Western blot

To obtain the protein sediment, cells were lysed in RIPA buffer at 4℃ for 30 min and centrifuged at 15,000 g for 15 min. Protein specimens were subsequently separated by SDS-polyacrylamide gel electrophoresis (SDS–PAGE) and transferred to PVDF membranes. The membranes were incubated with primary antibodies against Caspase-1 (1:1000 dilution), IL-1β (1:1,000 dilution), ASC (1:500 dilution), NLRP3 (1:500 dilution), GSDMD (1:1,000 dilution), IL-18 (10,663, 1:5000 dilution), and β-actin (1:5,000 dilution) overnight at 4 °C, followed by a 1-hour secondary antibody incubation (1:6,000 dilution) at room temperature. After washing, visualization was finally performed with ECL kit.

### Reactive oxygen species (ROS) detection

To detect cellular total ROS, DCFH-DA staining was carried out as previously described [20]. Treated and untreated cells were incubated with DCFH-DA staining solution (10 µmol/L) at 37 °C for 20 min. Subsequently, cells were washed three times with PBS to stop the reaction. Finally, images were captured using a fluorescence microscope, and the difference in fluorescence intensity was compared.

### Immunofluorescence

Fixed cells were permeabilized with 0.5% triton in PBS for 10 min followed by gently washing thrice. Then, cells were blocked with 3% BSA in PBST (0.1% triton in PBS) for 1 h at room temperature. Cells were subsequently incubated with the primary antibody (1:50 dilution) diluted in the blocking buffer for 2 h. After 3-times washing with PBST, cells were incubated with the secondary antibody (1:1,000 dilution) diluted in the blocking buffer for 1 h in the dark followed by staining of nuclei with DAPI for 5 min. The stained images were finally captured by the fluorescence microscope and analyzed by Image J software.

### Animal experiments

Male BALB/c nude mice (4-week-old) were purchanced from Hunan SJA Laboratory Animal Co., Ltd (Changsha, China). HCT116 cells (3 × 105 cells/200 µL/mouse) were subcutaneously injected into the left axilla of mice. After tumor formation, these mice were randomly divided into two groups, and given intragastric administration every day for three weeks with 0.9% saline (sham group) and 10 mg/kg simvastatin (simvastatin group). The tumor size was roughly measured twice a week. After 28 days, mice were sacrificed after intraperitoneal injection of 150 mg/kg pentobarbital sodium, and the isolated tumors were weighed and dissected into three parts for subsequent experiments. One part was fixed with formaldehyde and embedded by paraffin for hematoxylin & eosin (H&E) staining, another part was lysed with lysis buffer for western blot, and the other part were used to check ROS levels by DCFH-DA. For ROS detection, mixture of cells from two groups of tumors without DCFH-DA incubation served as the control. All operations involving animals were approved by the ethics committee of hunan cancer hospital (KYJJ-2022-233).

### Statistics

All the assays were performed in triplicate at least. Data were expressed as the mean ± standard error of mean (SEM) and analyzed with SPSS 13.0 software (IBM Corporation, Armonk, NY). Significant differences between groups were analyzed by t-test or one-way analysis of variance (ANOVA) followed by Duncan’s multiple comparison test, which were considered statistically significant with a *P* value < 0.05.

## Results

### Simvastatin repressed the cell proliferation in colon cancer cells

**Fig. 1:**
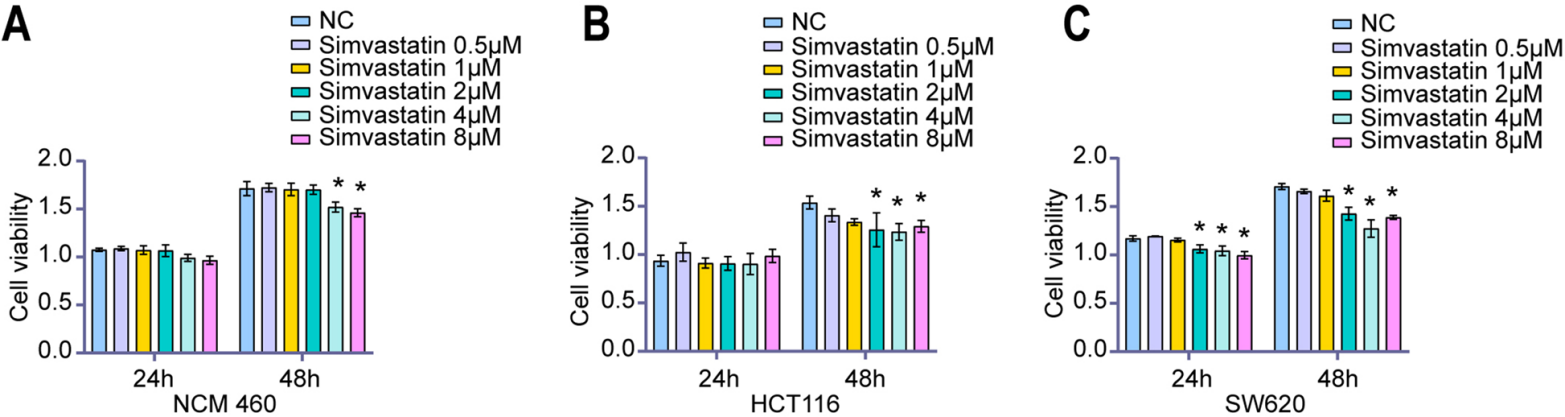
Simvastatin inhibits cell proliferation of HCT116 and SW620 cells. The viability of (**A**) NCM460, (**B**) HCT116 and (**C**) SW620 cells was detected by CCK-8 assay after exposure to different concentration of simvastatin

Inhibition of uncontrolled proliferation would be a potential target for cancer treatment. As demonstrated by the CCK-8 assay, simvastatin significantly inhibited NCM460, HCT116 and SW620 cell viability in a dose-dependent manner (Fig. 1). NCM460 cell proliferation were not suppressed until exposure to simvastatin at concentrations above 4 µM. Unlike normal colon epithelial cell, HCT116 and SW620 cell proliferation was significantly inhibited by simvastatin when the concentration was greater than 2 µM (Fig. 1A and B). Therefore, incubation of colon cancer cells with 2 µM simvastatin was used in subsequent analyses.

### Simvastatin triggered caspase-1 dependent pyroptosis via the canonical inflammasome pathway in colon cancer cells

**Fig. 2:**
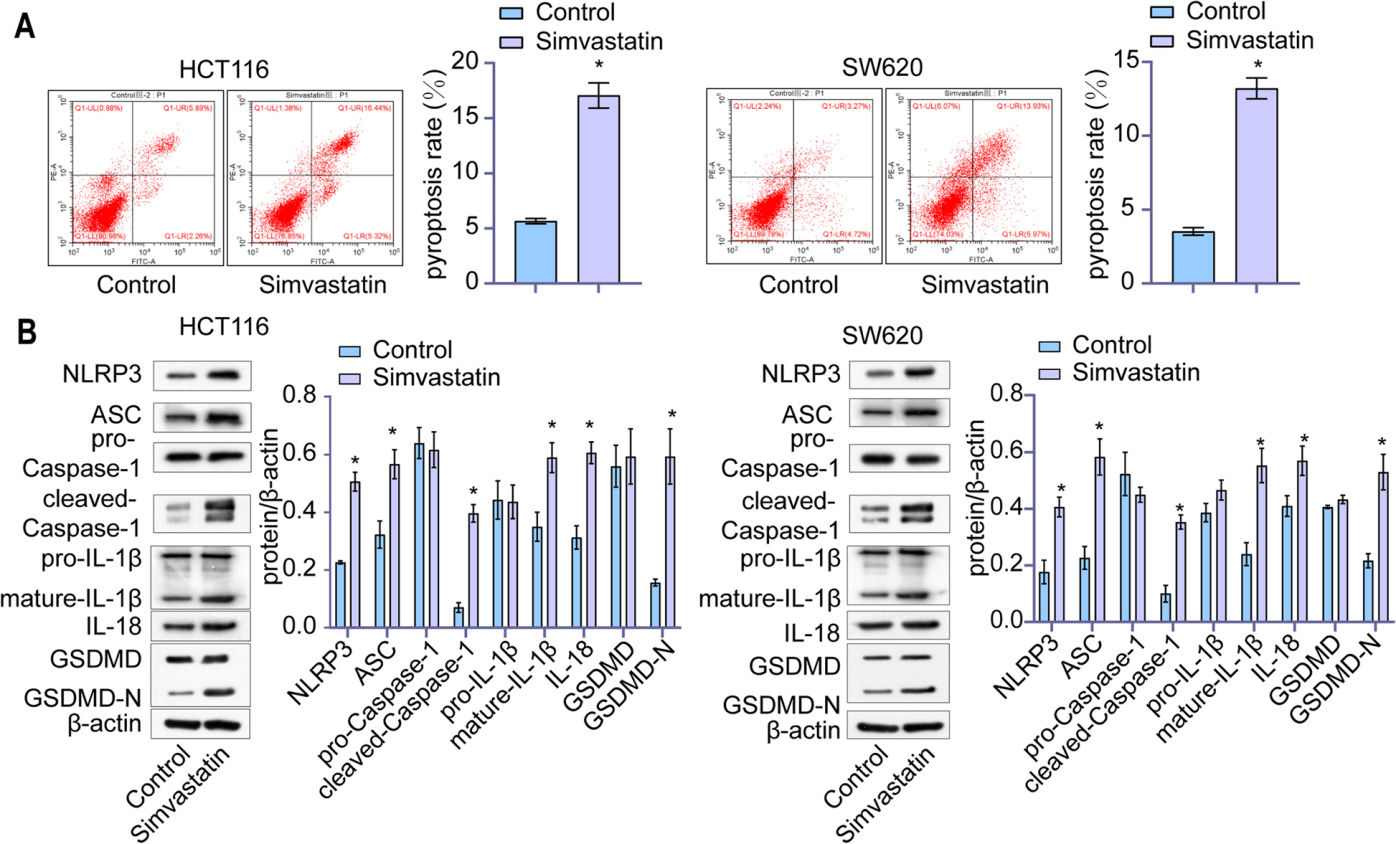
Simvastatin induced pyroptosis in HCT116 and SW620 cells. **A** After treating with simvastatin for 24 h, pyroptosis rate of cells was detected by flow cytometry analysis. **B** Levels of NLRP3, ASC, pro-caspase-1, cleaved-caspase-1, pro-IL-1β, mature-IL-1β, IL-18, GSDMD and GSDMD-N were detected by western blotting analysis in simvastatin treated and untreated cells, with β-actin as the reference protein. *, *p* < 0.05

To further decipher the effect of simvastatin on colon cancer cells, we investigated if simvastatin induced pyroptosis in colon cancer cells. As shown in Fig. 2A, the treatment of colon cancer cells with 2 µM simvastatin significantly increased pyroptosis rate by 2.94 and 4.15 times in HCT116 and SW620, respectively. Subsequently, we investigated the molecular pattern of treated and untreated colon cancer cells by qPCR and western blot assays. As shown in Supplemental Fig. 1, simvastatin increased cellular expression of NLRP3, ASC, caspase-1, GSDMD, IL-1β and IL-18 mRNA in HCT116 and SW620 cells. Although there was no differences detected in the total amount of pro-caspase-1, IL-1β, GSDMD, the levels of pyroptosis markers, cleaved-caspase-1, mature IL-1β and GSDMD-N significantly increased after simvastatin incubation. Furthermore, the expression level of caspase-1 upstream proteins, NLPR3 and ASC was significantly increased after exposure to simvastatin in colon cancer cells. Connected the key roles of NLPR3 and ASC with the canonical inflammasome pathway, these findings indicated that simvastatin incubation triggered pyroptosis via the canonical inflammasome pathway in colon cancer cells.

**Fig. 3:**
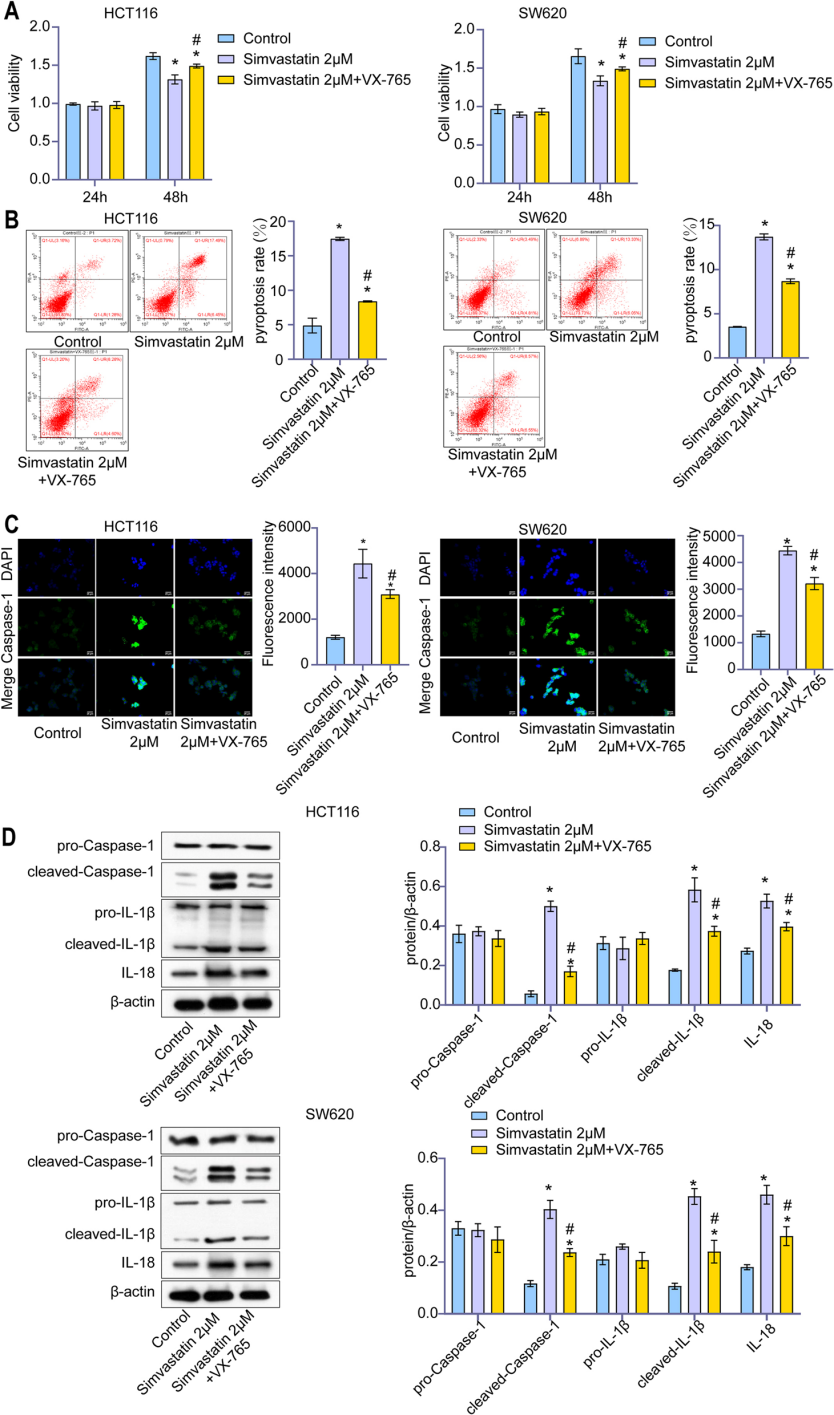
Simvastatin increased caspase-1 dependent pyroptosis in colon cancer cells. **A** The viability of HCT116 and SW620 cells was detected by CCK-8 assay after exposure to different drugs. **B** Pyroptosis rate of different groups of cells was detected by flow cytometry analysis. **C** Cells expressed activated caspase-1 were labeled by immunofluorescence staining. **D** Levels of pro-caspase1, cleaved-caspase1, pro-IL-1β, mature-IL-1β and IL-18 were detected by western blotting analysis, with β-actin as the reference protein. *, *p* < 0.05

To further validate the exact mechanism, we used VX-765 to inhibit Caspase-1 activity. As shown in Fig. 3A, VX-765 significantly reduced the inhibitory effect of simvastatin on colon cancer cell proliferation. As expected, VX-765 treatment partly rescued the simvastatin-mediated enhancement of pyroptosis rate in colon cancer cells (Fig. 3B). Then, we measured the activated caspase-1 in colon cancer cells by immunofluorescence staining. The proportion of cells expressing activated caspase-1 significantly increased after treatment with simvastatin, while VX-765 partly decreased this effect (Fig. 3C). To investigate the underlying molecular mechanism, we examined the expression level of caspase-1 downstream proteins. The results of western blotting indicated that VX-675 attenuated the induction effect of simvastatin on mature IL-1β and IL18 (Fig. 3D). It was in integration suggested that simvastatin induced caspase-1-dependent pyroptosis in colon cancer cells.

### Simvastatin regulated ROS to activate the canonical inflammasome pathway

**Fig. 4:**
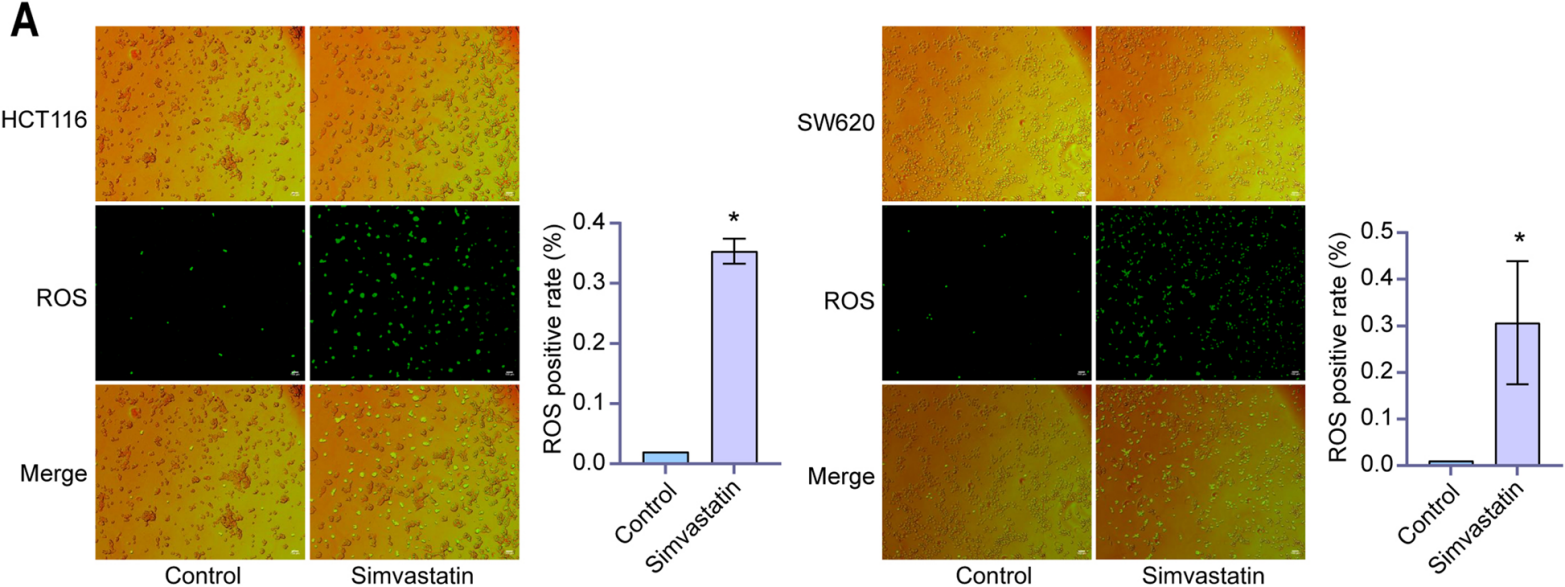
Simvastatin promotes cellular ROS production. Intracellular ROS were detected by DCFH-DA staining, and positive cells were calculated in HCT116 and SW620 cells

**Fig. 5:**
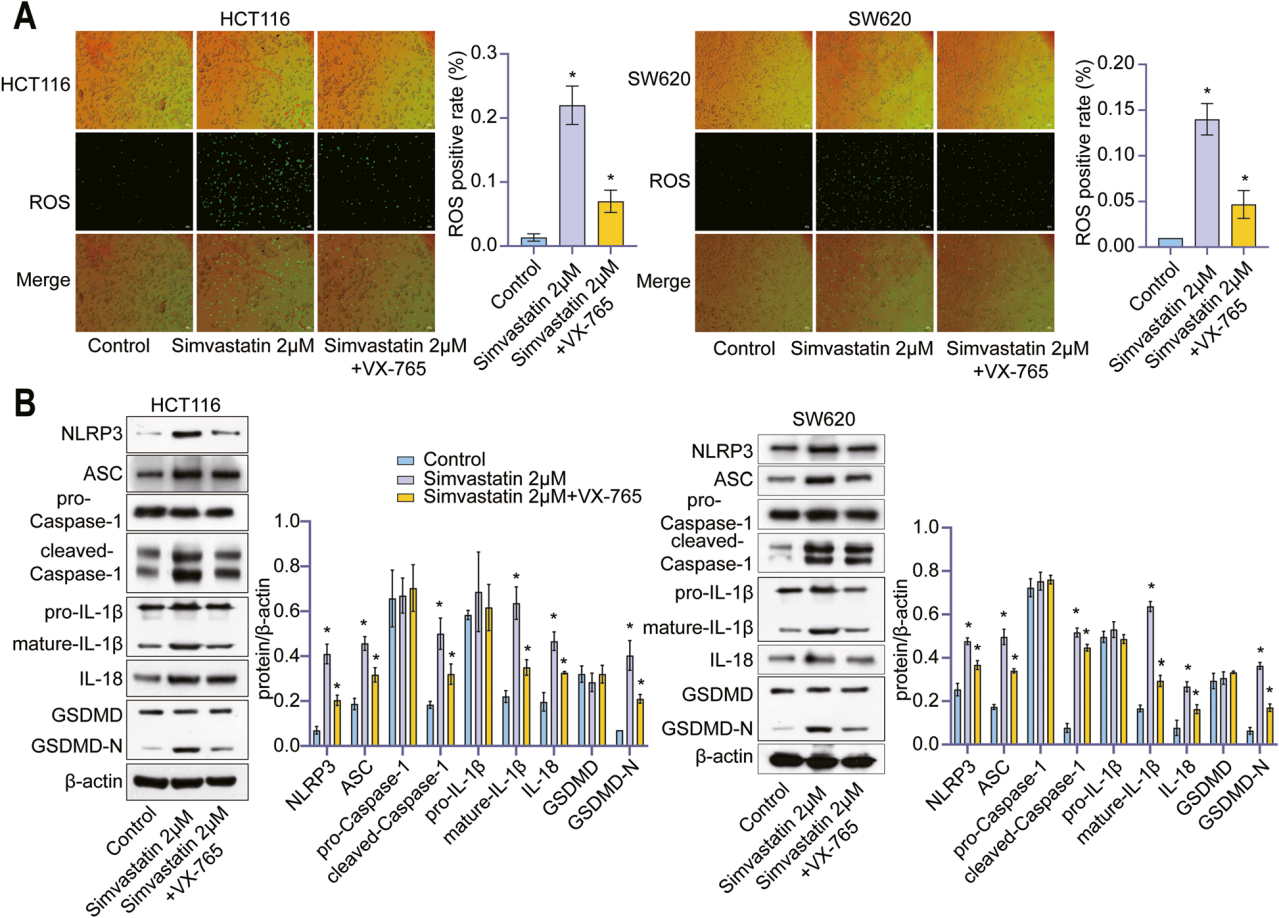
Simvastatin trigged colon cancer cell pyroptosis through activating ROS/NLRP3/Caspase-1 axis. **A** The rate of ROS-positive cells in different groups of cells. **B** Levels of NLRP3, ASC, pro-caspase1, cleaved-caspase1, pro-IL-1β, mature-IL-1β, IL-18, GSDMD, GSDMD-N were detected by western blotting analysis in different groups of cells, with β-actin as the reference protein. *, *p* < 0.05

To identify the simvastatin-triggered pyroptosis pathway, we measured intracellular ROS production as ROS release triggered formation of the NLRP3 inflammasome. As shown in Fig. 4, Simvastatin induced intracellular ROS production in the HCT116 and SW620 cells. To further validate the role of ROS in simvastatin-mediated pyroptosis, we used NAC to antagonize ROS. After pre-treatments, NAC effectively quenched the ROS-specific signal induced by simvastatin in colon cancer cells (Fig. 5A), suggesting that blockade of ROS signal inhibited simvastatin-induced cell pyroptosis. Moreover, the results of western blotting indicated that NAC effectively rescued the induction effect of simvastatin on NLRP3/caspase-1 pathway and pyroptosis biomarkers (Fig. 5B).

**Fig. 6:**
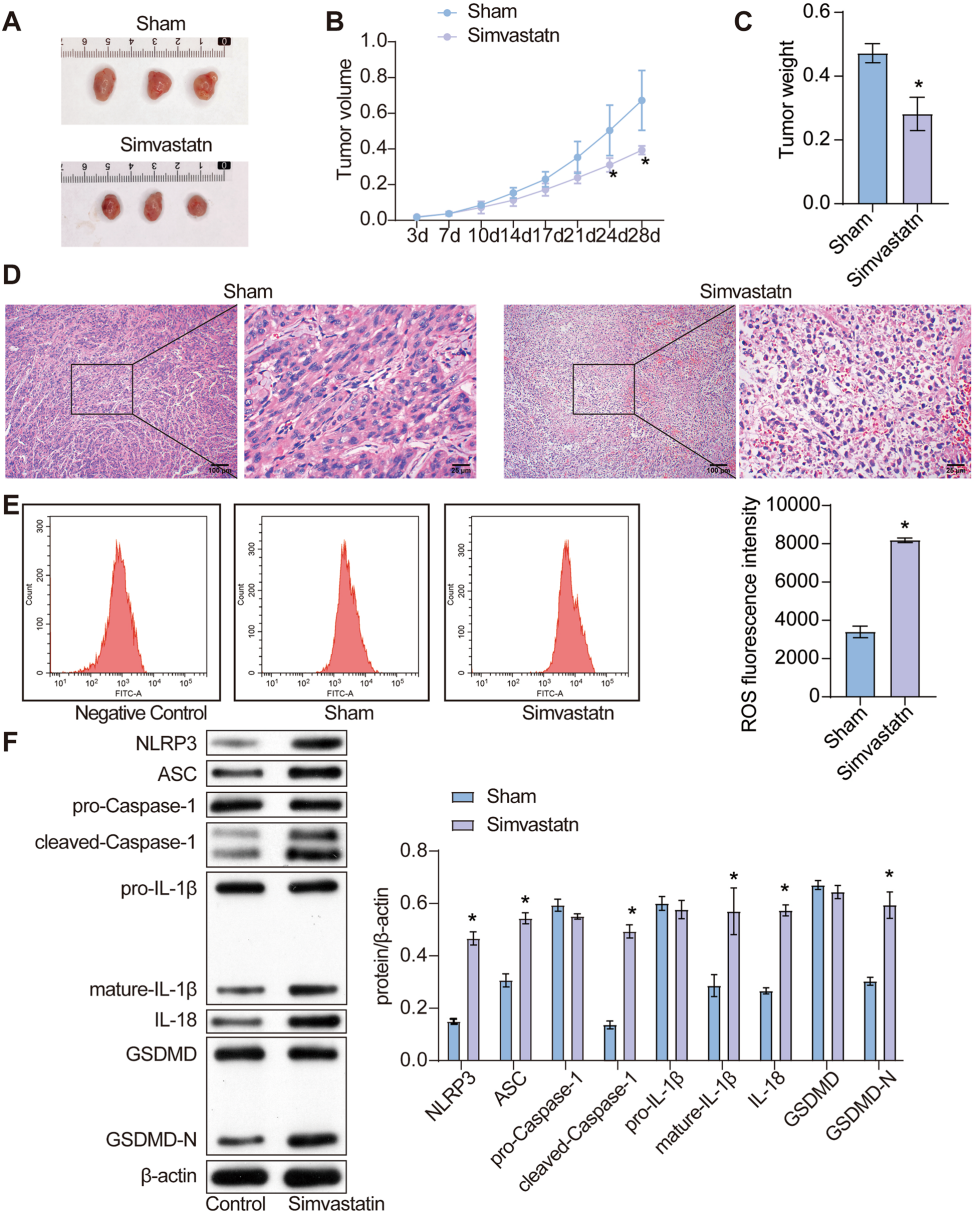
Simvastatin inhibits pyroptosis and tumor growth in vivo. **A** Representative photographs of isolated tumors in each group. The tumor tissues from two rows were taken from sham group and simvastatin group, respectively. **B** The tumor growth curve of each treatment group was presented in line charts. The volume of tumors from mice of each group was measured and recorded twice a week for 4 weeks. **C** The weight of isolated tumors in each group was presented in bar charts. **D** Tumor tissues were fixed with formaldehyde and embedded by paraffin for H&E staining. **E** Levels of ROS in isolated tumors were detected by using DCFH-DA probe and flow cytometry at day 28 after treatment. **F** levels of NLRP3, ASC, pro-Caspase-1, cleaved-Caspase-1, pro-IL-1β, mature-IL-1β, IL-18, GSDMD, GSDMD-N were tested in tumors from mice of each group, with β-actin as the reference protein. *, *p* < 0.05

To further study the effects of simvastatin on tumor growth in vivo, the tumor-bearing mice were treated with 10 mg/kg simvastatin. Compared with the sham group, tumor size (Fig. 6A and B) and weight(Fig. 6C) of the test group obviously decreased. Futhermore, it was demonstrated in the data of H&E staining that simvastatin treatment in vivo enhanced cell death (Fig. 6E). Flow cytometry analysis showed that simvastatin treatment resulted in significantly higher efficiency of ROS production in isolated tumors (Fig. 6F). In addtion, the protein level of NLRP3, ASC, cleaved-caspase-1, mature IL-1β and GSDMD-N in isolated tumors significantly increased after simvastatin treatment, suggesting the induction of simvastatin on pyroptosis in colon cancer in vivo.

## Discussion

Statins were formerly identified as a class of drugs used for dyslipidemia treatment. Recently, an increasing evidence supports the anti-cancer activity of this class of drugs; however, the underlying mechanisms have not been well characterized. In the present study, we found simvastatin induced pyroptosis and thereby repressed the cell proliferation of colon cancer cells.

Statins are beneficial for cardiovascular health and prevent coronary heart disease; therefore, their anticancer properties have received more and more attention [21]. For instance, fluvastatin was reported to be an effective agent for preventing metastasis of renal cancer [22]. Xiao’ study indicated that lovastatin suppressed the canonical Wnt/β-catenin and alternative Wnt-YAP/TAZ pathways via inhibiting RhoA activity in colon cancer cells [23]. In addition, it has been reported that simvastatin and atorvastatin inhibit the proliferation of human cholangiocarcinoma cells [3]. In our study, we found that simvastatin could repress colon cancer cell proliferation in vitro, which was consistent with previous studies referring to the anti-cancer activities of statins. Besides, our findings indicated that simvastatin inhibited the growth of colon cancer cell through inducing pyroptosis. Previous studies regarded the induction of apoptosis as a reason for the anti-cancer effect of simvastatin [24, 25]. In the present study, we found that simvastatin treatment induced pyroptosis in colon cancer cells, which might be a significant addition to the previous studies. Some bioinformatics studies indicated that proptosis-related gene signature might be help in making therapeutic decisions for patients with colon cancer [26, 27]. As a result, it was suggested the essential role of pyroptosis in colon cancer. Moreover,, we examined the expression of pyroptosis-related biomarkers to further study the mechanism of simvastatin-induced pyroptosis by suppressing some key components in the pathway. The caspase-1 specific inhibitor, VX-765, has been reported to show protective effect in colorectal cancer [28, 29]. Although previous study indicated the importance of caspase-3/GSDME pyroptosis pathway in colon cancer [30–32], our present study found simvastatin-mediated pyroptosis decreased in colon cancer cell when caspase-1 activity was inhibited by VX-765 incubation. This NLRP3/caspase-1/GSDMD pyroptosis pathway in response to simvastatin in colon cancer cells was consistent with the previsous study in non-small cell lung cancer [17]. However, research to date had not yet determined the signaling between simvastatin and NLRP3-containing inflammasome complex. Previous studies indicated that simvastatin induced the intracellular accumulation of ROS [4, 33], who always acted as a second messenger in cell signaling. Accordingly, we used NAC to block ROS and investigated changes in the downstream signaling. Our results indicated that simvastatin-induced pyroptosis was suppressed after blocking cellular ROS, suggesting that simvastatin activated NLRP3-containing inflammasome complex through increased cellular ROS. Although we provide some novel insights into the underlying mechanism of simvastatin in colon cancer treatment, there are some limitations in the present study. The findings of this study were derived solely from colon cancer cell lines and cell line-based xenograft tumor models, and thus using more elaborate cancer models, such as organoid [34, 35], spheroid [36, 37] and patient-derived xenograft [38] models, might make the conclusion more convincible. Moreover, clinical studies could be further designed to demonstrate the response of colon cancer patients to simvastatin. Besides, subsequent in-depth studies can be continued to explore novel simvastatin-containing combination strategies for the treatment of colon cancer.

## Conclusions

In conclusion, we have revealed that simvastatin induces pyroptosis through ROS/NLRP3/caspase-1/GSDMD pathway in colon cancer. Our findings provide novel insights into future understanding with the mechanism of simvastatin in colon cancer treatment.

### Supplementary Information

**Additional file 1: Figure S1.** Simvastatin increase mRNA expression of pyroptosis related genes. Levels of NLRP3, ASC, caspase-1, GSDMD, IL-1β and IL-18 were detected by qPCR analysis in simvastatin treated and untreated cells, with GAPDH as the reference protein. *, *p* < 0.05

## Data Availability

Data will be available upon reasonable request from the corresponding author.

## Abbreviations

ROS: Reactive oxygen species
PCD: Programmed cell death
CCK-8: Cell counting kit-8
PI: Propidium iodide
SDS–PAGE: SDS-polyacrylamide gel electrophoresis
ECL: Enhanced chemiluminescence
H&E: Hematoxylin & eosin
SEM: Standard error of mean
ANOVA: Analysis of variance
