# Supplementary figures and images for: Simvastatin induces pyroptosis via ROS/caspase-1/GSDMD pathway in colon cancer
Source: Cell Commun Signal. 2023 Nov 16;21:329. doi: 10.1186/s12964-023-01359-y (PMC10652480; doi:10.1186/s12964-023-01359-y)

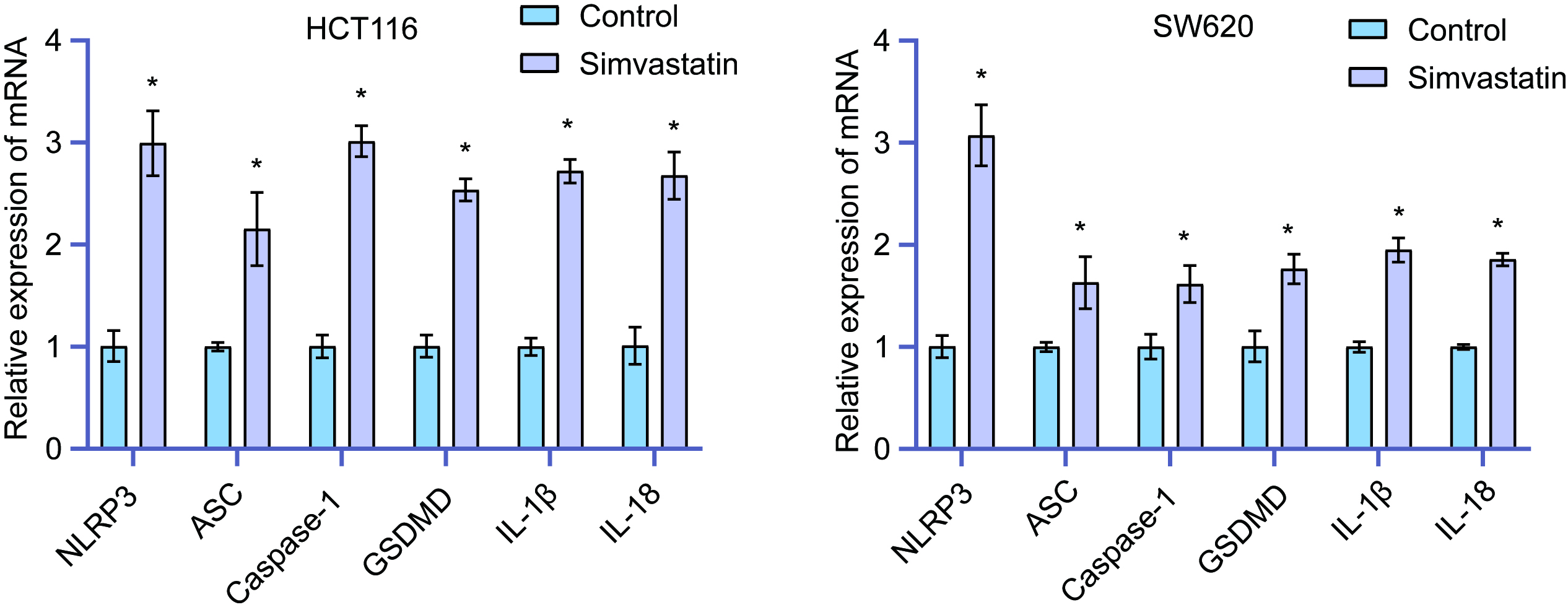

Supplement: Supplementary file 2 — Additional file 1: Figure S1. Simvastatin increase mRNA expression of pyroptosis related genes. Levels of NLRP3, ASC, caspase-1, GSDMD, IL-1β and IL-18 were detected by qPCR analysis in simvastatin treated and untreated cells, with GAPDH as the reference protein. *, p < 0.05 [file 12964_2023_1359_MOESM1_ESM.jpg]
